# Arthroscopic Removal of a Free-Floating Bullet From the Hip Joint

**DOI:** 10.7759/cureus.51127

**Published:** 2023-12-26

**Authors:** Alexandros Tzaveas, Theofylaktos Kyriakidis, Vasileios Chouliaras, Michael Iosifidis

**Affiliations:** 1 3rd Orthopedic Department, Interbalkan Medical Center, Thessaloniki, GRC; 2 Orthopedic Surgery Department, General Hospital of Arta, Arta, GRC

**Keywords:** hip, war trauma, hip injury, bullet extraction, gunshot injury, hip arthroscopy

## Abstract

Gunshot injuries and bullet removal are extremely rare indications for hip arthroscopy. We present the case of a 22-year-old male with a free-floating bullet in the hip joint after a gunshot injury. A thorough imaging investigation was used to demonstrate the exact location of the foreign body. The bullet was removed by arthroscopic means under fluoroscopic guidance. The patient has been symptom-free for two years postoperatively. The tips and tricks of the technique are discussed. Hip arthroscopy is a minimally invasive technique to remove a free-floating bullet and avoid potential long-term complications like chondral injury and lead intoxication.

## Introduction

Hip arthroscopy has been extensively utilized in the last 20 years, with the main indications being femoroacetabular impingement and acetabular labral tears [1]. Technical advancement and an increase in surgeons' experience led to the broadening of indications [2]. However, trauma cases treated arthroscopically have been reported only sporadically in the literature. Especially for cases with free-floating bullets in the hip joint, hip arthroscopy yields superiority to open arthrotomy, offering the major advantage of foreign body removal and washout with minimal surgical procedure and quicker recovery [3]. In addition, the risk of complications and potential dislocation has been decreased [2]. We report the arthroscopic removal of a bullet from the hip joint, the preoperative imaging examination, some technical pearls, and the postoperative results.

## Case presentation

A 22-year-old Libyan soldier was transferred to our unit after he sustained a gunshot injury two months ago. The entrance wound was located in the left lower abdominal area, and the bullet caused trauma to the left femoral nerve and femoral artery and then landed in the right hip. His left femoral artery trauma had been treated elsewhere and then transferred to our unit. He underwent nerve reconstruction by the neurosurgeons. No other splanchnic injuries were found in the pelvic cavity. After initial postoperative mobilization, he developed right hip pain and difficulty in waking; thus, removal of the bullet from the right hip was decided in order to avoid potential septic arthritis, wear of the joint, and lead intoxication.

**Figure 1:**
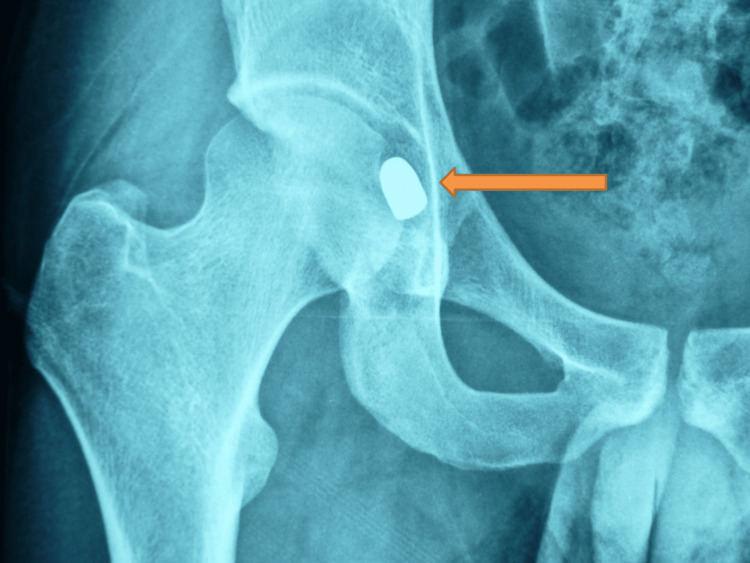
Plain anteroposterior radiograph of the right hip Arrow indicates the free-floating bullet located in the hip joint

**Figure 2:**
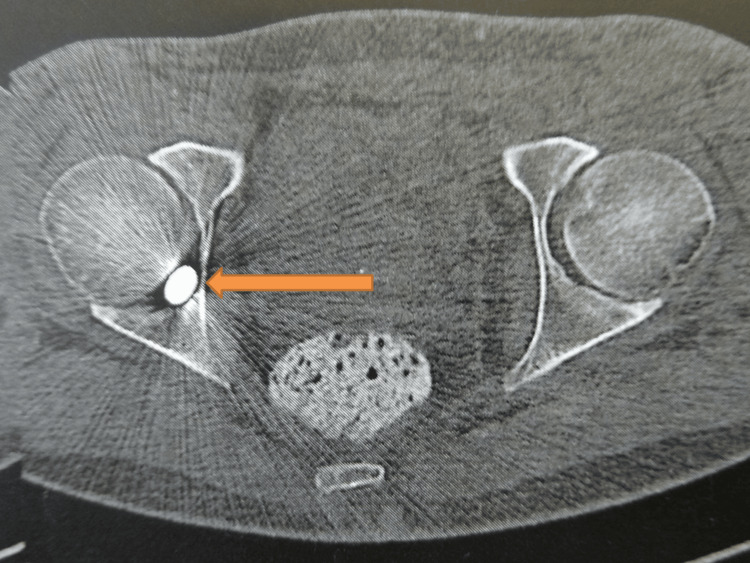
Axial section of the CT scan of the hips Arrow indicates the exact location of the bullet in the cotyloid fossa

The patient underwent radiographic and CT examinations in order to specify the exact location of the bullet and its anatomic relations with the other joint structures (Figures 1-2). On the plain radiograph, the bullet was found to be free-floating in the hip joint. Examination with a CT scan revealed the bullet to be in the cotyloid fossa. As the intra-articular position was established with no obvious fracture or osseous deficit, a decision was made to proceed with arthroscopic exploration and removal. No other fragments or foreign bodies were identified. The patient consented for the procedure and, additionally, for potential open dislocation and arthrotomy in the event of an inability to remove the bullet.

**Figure 3:**
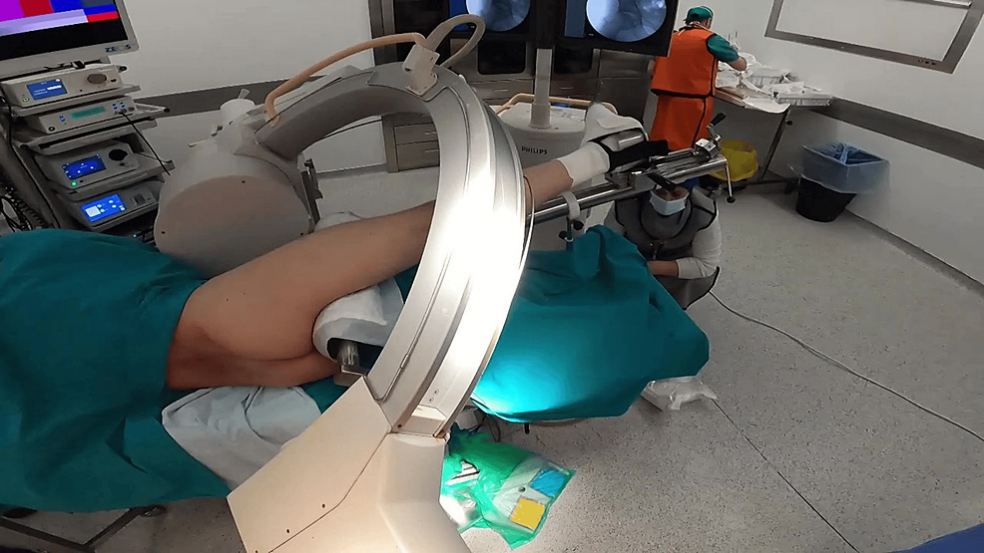
Theatre set-up for hip arthroscopy Patient positioned in the lateral position with a special traction device. Fluoroscopy (C-arm) was used throughout the whole operation

**Figure 4:**
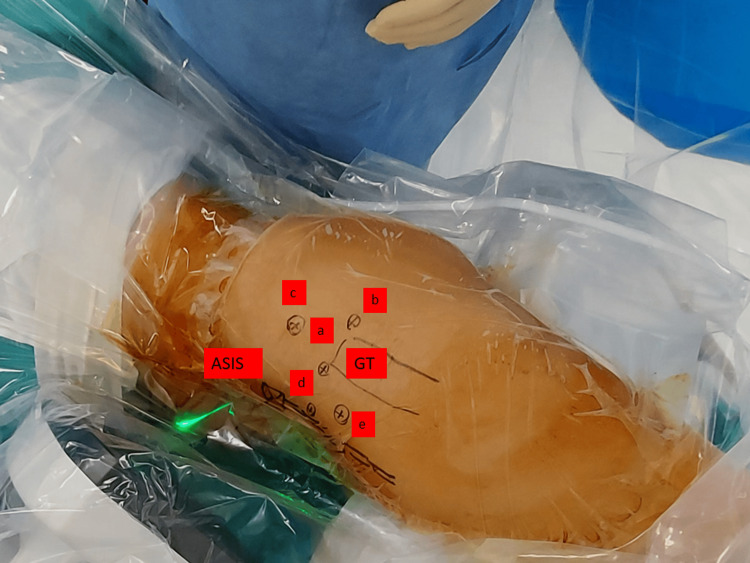
Arthroscopic portals Portals and anatomic landmarks: (ASIS) anterior superior iliac spine, (GT) greater trochanter, (a) anterolateral portal, (b) posterolateral portal, (c) supratrochanteric portal, (d) anterior portal, (e) mid-anterior portal

**Figure 5:**
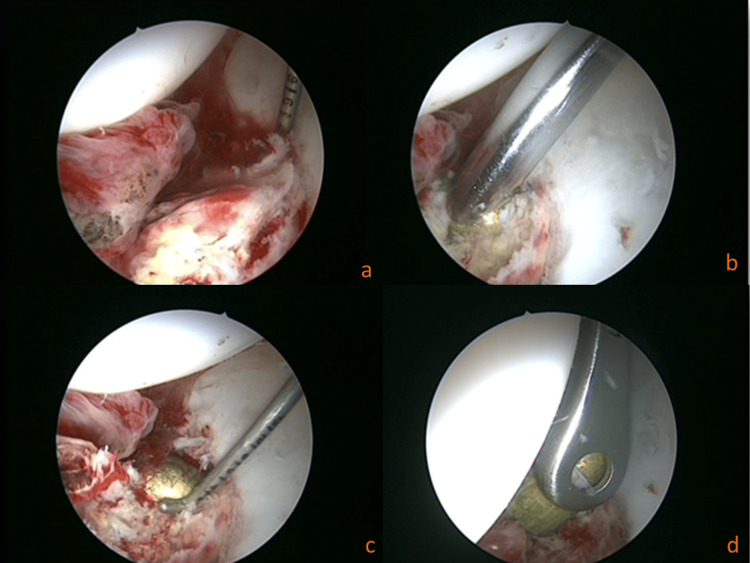
Arthroscopic views of the right hip The sequence of manoeuvers and extraction of the bullet from the cotyloid fossa: (a) bullet hidden under the soft tissues, (b) shaving reveals the bullet, (c) mobilization with the probe, (d) grasping with the pituitary rongeur

**Figure 6:**
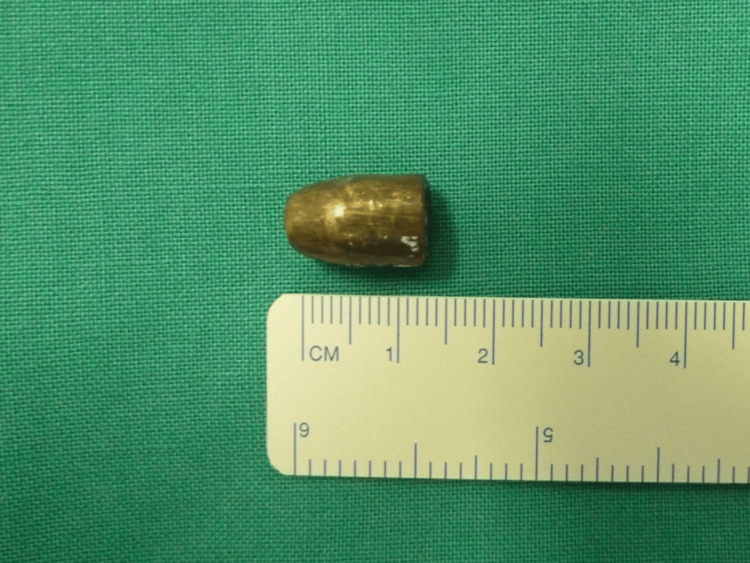
Macroscopic view of the bullet Bullet measuring approximately 15 x 7 millimeters

The patient was positioned in the lateral decubitus position with a special traction device. Hip arthroscopy was undertaken using anterolateral and posterolateral portals for the central compartment and a supratrochanteric for the peripheral compartment (Figures 3-4). After traction, access to the joint was established with a 70-degree arthroscope. Upon access, joint aspirations were made for cultures and sensitivities. A complete exploration of the central compartment was done, observing the anterior and posterior surfaces of the femoral head, anterior and posterior acetabulum, acetabular labrum, and ligamentum teres. No chondral damage or acetabular labral tears were observed. The bullet was not found in the first place, and an image intensifier was used to find its exact position in the cotyloid fossa. The posterolateral portal was used as a viewing portal and the anterolateral as a working portal in order to gain better access to the area of the cotyloid fossa. Using an arthroscopic shaver, the area of the pulvinar was cleared, and by tactile sensation and image intensifier guidance, the bullet was exposed and revealed by further shaving of the area (Figure 5). Pushing and twisting maneuvers were used to mobilize it. Further traction was used to increase the space between the femoral head and the acetabulum due to the large size of the bullet. Finally, it was removed with a pituitary rongeur. Fluoroscopy confirmed bullet removal (Figure 6). The joint was then irrigated with 4 liters of saline. After traction release, additional access was gained also to the peripheral compartment to check for other potential pathologies, and further irrigation was undertaken. The fluid pressure was kept at 50 mmHg for the whole procedure. After the end of the operation, the patient’s abdomen remained soft. Postoperatively, cephalosporin was administered intravenously until culture results showed no infection. The patient mobilized immediately after the operation with partial weight bearing. He had an uneventful stay in the hospital and was discharged in 48 hours. He had a physiotherapy course for six weeks. He returned immediately to everyday activities and remained symptom-free two years after the operation.

## Discussion

It has been shown that intraarticular, free-floating bullets may cause chondral injury, septic arthritis, or even lead intoxication [4-6]. Chondral injury and septic arthritis may predispose to early joint destruction and osteoarthritis. Lead intoxication results from systemic absorption from the synovial fluid and may lead to multisystem disease. As these are catastrophic complications, bullets must be removed from the joints.

Hip exploration by open means requires a very traumatic approach with a large incision, potential trochanteric osteotomy, and ligamentum teres tear, and carries the risk of nervous and vascular injury, avascular necrosis of the femoral head, trochanteric pseudoarthrosis, and heterotopic ossification [7]. On the other hand, hip arthroscopy has evolved significantly in the last few years, and surgeons’ expertise has grown; as such, it is an efficient alternative technique for such patients. Nevertheless, hip arthroscopy carries also the possibility of complications, with the extravasation of fluid in the abdominal cavity being the most severe, though very rare [8].

Management of patients with free-floating bullets in the hip requires a thorough imaging evaluation to ensure that there is no major fracture, especially at the acetabular side, and also to precisely depict the bullet position. Traction should allow a complete round of the joint and should create an efficient space between the femoral head and acetabulum for bullet removal. All portals need to be available for approaching throughout the joint. A “hidden” bullet may need fluoroscopy for identification, and palpation with the arthroscopic probe may help the surgeon locate it. A wide variety of graspers and a pituitary rongeur need to be available for grasping the bullet. The working portal may need enlargement to allow bullet extraction. Fluid pressure should remain relatively low to avoid extravasation of fluid.

To the best of our knowledge, there are only 14 published cases of intraarticular bullets removed either purely arthroscopically or assisted with some incision. However, only six cases with free-floating bullets are included. Goldman et al. [9] were the first to publish arthroscopic bullet removal from the hip, assisted by a mini-incision. Cory et al. [10] removed an arthroscopic bullet from the femoral head together with osteochondral loose bodies. Teloken et al. [11] also removed a bullet lodged in the femoral head, assisted by a mini-incision. Mineo et al. [12] and Gupta et al. [13] removed arthroscopically a bullet embedded in the acetabulum, and Singleton et al. [14], on a similar occasion, used a drill bit. Sozen et al. [15], Kaya et al. [16], and Catma et al. [17] removed free-floating bullets arthroscopically. Al-Asiri and Wong [18], Gurpinar and Ozturkmen [19], and Ferro et al. [5] removed bullets embedded in the acetabulum, whereas Howse et al. [20] reported seven cases, of which four were treated exclusively by arthroscopy, with one having a free-floating bullet, and Mullis et al. [8] published a series of eight patients, seven of whom had loose bodies and one removal of a whole bullet.

## Conclusions

We herein report a rare case of an intraarticular bullet of the hip joint after a gunshot injury that was removed arthroscopically. Careful preoperative imaging is of paramount importance for an accurate assessment of bullet location. Removal by arthroscopic means requires hip arthroscopy skills; however, with the use of certain tips, an excellent postoperative result may be achieved.
